# Intrinsic Coupling of Radiative Cooling and Triboelectric Responses in Dual‐Function ZrO_2_ Nanocomposites for Adaptive Thermoregulation

**DOI:** 10.1002/advs.76121

**Published:** 2026-06-15

**Authors:** Yoon Young Choi, Pranto Karua, Md Salauddin, Lili Cai

**Affiliations:** ^1^ Department of Mechanical Science and Engineering the Grainger College of Engineering University of Illinois Urbana‐Champaign Urbana Illinois USA; ^2^ Materials Research Laboratory the Grainger College of Engineering University of Illinois at Urbana−Champaign Urbana Illinois USA

**Keywords:** direct ink writing, energy harvesting, radiative cooling, thermoregulation, zirconium oxide

## Abstract

Efficient integration of radiative cooling and triboelectric function remains challenging due to the decoupled optimization of optical and electrical properties in conventional systems. Here, we report a nanocomposite design strategy enabled by zirconium oxide (ZrO_2_) that intrinsically couples passive daytime radiative cooling and triboelectric functionality within a single material platform. Leveraging its high refractive index and dielectric polarization capability, ZrO_2_ simultaneously enhances broadband solar scattering and triboelectric charge generation without multilayer integration. This unified material system is processed via direct ink writing (DIW) to fabricate fiber‐structured architecture with precise geometric control, while preserving breathability, washability and flexibility. The resulting DIW‐printed textile exhibits a high solar reflectance of 96% and strong mid‐infrared emissivity of 97%, achieving sub‐ambient cooling of 3°C–6°C under direct sunlight. In addition, the textile delivers a peak power density of 47 mW m^−2^ and maintains stable electrical output over 30 000 operating cycles while operating as a triboelectric nanogenerator. This textile platform further enables motion‐controlled thermoregulation, in which triboelectric signals generated from biomechanical activity directly trigger on‐demand Joule heating, allowing seamless switching between passive cooling and active heating under dynamic conditions. These results provide a generalizable pathway toward adaptive, multifunctional material systems for wearable technology and beyond.

## Graphical abstract

A novel nanocomposite design strategy utilizes ZrO_2_ to intrinsically couple passive daytime radiative cooling and triboelectric functionality within a single material platform. Fabricated via direct ink writing, the multifunctional breathable architecture provides effective sub‐ambient cooling, motion‐driven energy harvesting, and adaptive thermoregulation, establishing an integrated strategy for wearable thermal management systems.

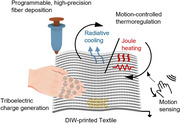

## 1 Introduction

Efficient personal thermal management remains a major challenge for next‐generation wearable technologies [1, 2, 3]. Conventional air‐conditioning and heating systems are stationary, energy‐intensive, and impractical for personal use, underscoring the need for lightweight wearable materials capable of providing localized thermal regulation with minimal energy consumption [4, 5, 6, 7]. Passive daytime radiative cooling (PDRC) offers a promising solution for sustainable thermal comfort by reflecting incoming solar radiation while emitting mid‐infrared body heat through the atmospheric transparency window [8, 9, 10, 11, 12, 13, 14, 15, 16, 17]. Owing to its passive and continuous cooling capability, PDRC has been widely explored in recent years for personal comfort and energy‐saving applications [5, 18, 19, 20, 21, 22, 23, 24, 25, 26, 27, 28, 29, 30].

In parallel, textile‐based triboelectric nanogenerators (TENGs) have emerged as an attractive approach for harvesting biomechanical energy and enabling self‐powered sensing [31, 32, 33, 34, 35]. By converting human motion into electricity, TENGs can power small electronic devices and provide real‐time physiological and gesture sensing, making them highly appealing for wearable electronics and human–machine interfaces [36, 37, 38, 39, 40]. Recent efforts have sought to integrate PDRC and TENG functionalities into multifunctional wearable systems that combine thermal regulation, energy harvesting, and sensing [41, 42, 43, 44, 45, 46]. While these studies represent important progress, existing approaches typically rely on multilayer assemblies or hybrid system integration, in which optical and electrical components are designed independently and subsequently combined [46, 47, 48]. Such strategies inherently introduce trade‐offs between optical performance, electrical output, and mechanical compliance, limiting the ability to simultaneously optimize these functionalities within a single material platform.

A fundamental challenge therefore lies in developing materials that can intrinsically couple optical and dielectric responses, enabling simultaneous control over radiative and triboelectric properties through unified nanoscale design. In particular, achieving high solar reflectance for radiative cooling and strong dielectric polarization for triboelectric charge generation typically requires distinct material characteristics, making their integration within a single system nontrivial. Overcoming this limitation requires a materials‐level strategy that bridges electromagnetic interactions across optical and electrostatic regimes.

In this work, we report a nanocomposite design strategy enabled by zirconium oxide (ZrO_2_) that intrinsically couples radiative cooling and triboelectric functionalities within a single material system. Leveraging its high refractive index and dielectric properties, ZrO_2_ simultaneously enhances broadband solar scattering and dielectric polarization, enabling concurrent optimization of optical and electrical performance. This dual‐role behavior establishes a unified mechanism for coupling radiative and triboelectric responses, eliminating the need for multilayer integration. This unified material platform further enables additive manufacturing via direct ink writing (DIW) to fabricate fiber‐structured architectures with precise geometric control. The DIW‐defined textile structure establishes a direct linkage between nanocomposite composition, microstructural organization, and macroscopic multifunctional performance, while preserving breathability and mechanical compliance. Importantly, this architecture enables synergistic coupling between biomechanical motion and thermal regulation, where motion‐induced triboelectric signals directly trigger on‐demand thermal response, enabling intelligent, activity‐responsive personal thermal control. This work establishes a general materials and structural design framework for intrinsically coupled multifunctional systems, in which nanoscale dielectric engineering enables simultaneous control of optical, electrical, and thermal behaviors. The resulting platform demonstrates a pathway toward adaptive, energy‐autonomous materials for wearable systems and beyond.

## 2 Results and Discussion

### 2.1 Design and Architecture of the PZO Textile

**FIGURE 1:**
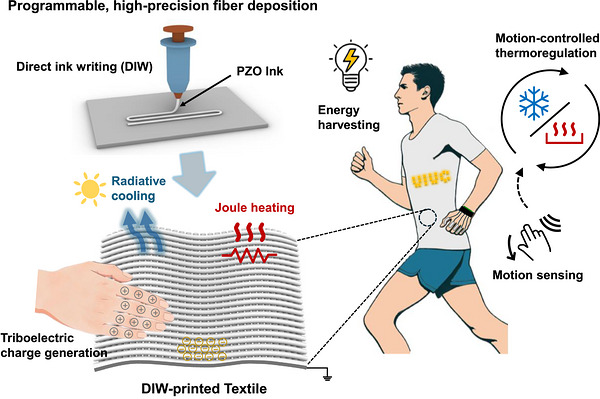
Schematic illustration of the DIW‐printed multifunctional textile, depicting the direct ink writing process, fiber‐structured textile design for passive daytime radiative cooling, integrated triboelectric charge generation functionality for energy harvesting and sensing, and motion‐triggered Joule heating enabled by the underlying conductive textile layer.

Figure 1 illustrates the design concept of the multifunctional textile fabricated by DIW of a polydimethylsiloxane–zirconium oxide (PDMS‐ZrO_2_, PZO) nanocomposite, which intrinsically integrates PDRC and TENG within a fiber‐structured textile architecture. Unlike conventional coated or laminated textiles, DIW enables programmable deposition of the nanocomposite into a conformal and breathable textile with precisely defined fiber geometry. Within this architecture, ZrO_2_ serves a dual function, with its high refractive index promoting strong solar reflection for efficient radiative cooling and its dielectric polarization capability amplifies triboelectric output within the PDMS matrix. Meanwhile, the PDMS phase provides strong mid‐infrared emissivity, enabling efficient thermal radiation through the atmospheric window. This approach embodies a collaborative design where the optical and dielectric requirements are addressed simultaneously by a single material, rather than being decoupled into separate functional layers. By leveraging both the high refractive index and strong dielectric polarization of ZrO_2_ within a single nanocomposite matrix, the system achieves concurrent optimization of radiative cooling and triboelectric performance.

Beyond radiative cooling, the DIW‐printed PZO textile functions as a triboelectric layer that converts mechanical motion into electrical energy via contact‐separation with the skin. The resulting triboelectric signals can be harvested to power small wearable devices or utilized for real‐time motion and gesture sensing. Notably, the fiber‐structured geometry enhances conformal contact and mechanical compliance, contributing to stable triboelectric performance under repeated deformation. When combined with a conductive textile substrate, the platform enables dual‐mode thermal management, maintaining passive cooling under sunlight and activating motion‐triggered Joule heating in cold conditions. This DIW‐enabled textile design establishes a unified platform that synergistically integrates energy harvesting, motion sensing, and adaptive thermal regulation within a single, mechanically compliant structure, while maintaining simplicity and scalability for wearable applications.

**FIGURE 2:**
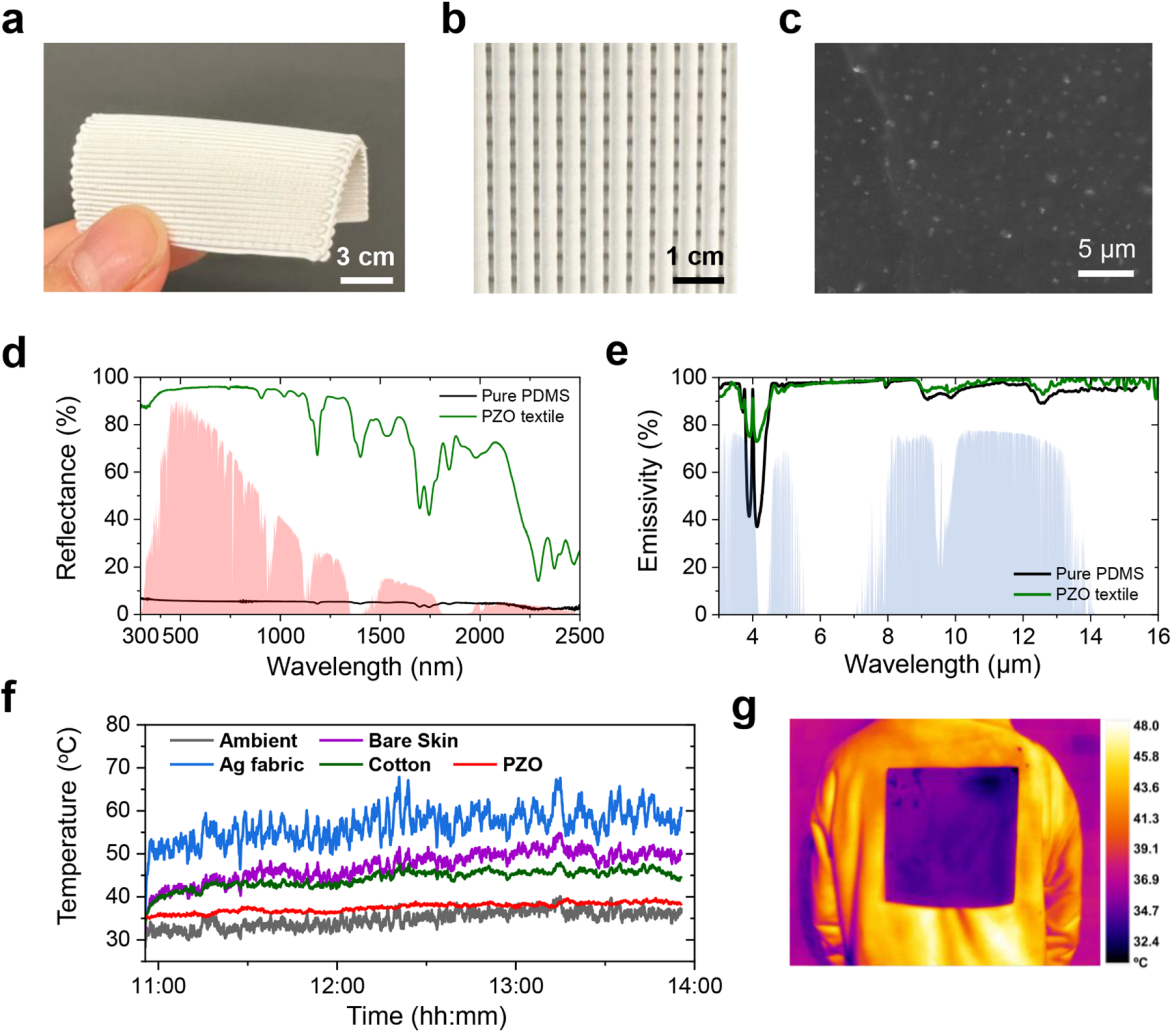
Optical and thermal characterization of the DIW‐printed PZO textile. Photographs demonstrating (a) its mechanical flexibility and (b) permeable fiber‐structured geometry. (c) Cross‐sectional SEM image of a PZO fiber. (d) Solar reflectance and (e) mid‐infrared emissivity of the printed PZO textile, compared with pure PDMS. Shaded areas indicate the AM1.5G solar spectrum (red) and the atmospheric transmission window (blue). (f) Outdoor temperature measurements under direct sunlight comparing the PZO textile with bare skin, cotton fabric, and Ag‐coated fabric. (g) Infrared thermal image of a large‐area printed PZO textile worn on the back of a shirt under sunlight exposure.

Figure 2a demonstrates the flexibility of DIW‐printed fiber textile, which can withstand repeated bending and deformation without mechanical failure or loss of structural integrity. This robustness arises from the compliant polymer matrix combined with a continuous, interconnected fiber network. Moreover, the PDMS‐based matrix is known for its excellent environmental durability and chemical stability, with previous studies reporting negligible mass loss and stable physicochemical performance under prolonged operating conditions, supporting the suitability of the DIW‐printed textile for long‐term wearable applications [20, 41]. A magnified optical image in Figure 2b further reveals the well‐defined fiber geometry and ordered textile architecture enabled by DIW, which facilitates excellent breathability. As shown in Figure S1, the printed textile exhibits a water vapor permeation rate of 4.12 mg·cm^−^^2^·h^−^^1^, comparable to that of commercial cotton fabric 4.75 mg·cm^−^^2^·h^−^^1^, indicating breathable performance comparable to commercial textiles. In addition, the fiber network displays a uniform white appearance, indicative of strong diffuse solar reflection. This optical behavior is attributed to the rational dispersion of ZrO_2_ nanoparticles within the polymer matrix, as evidenced by the scanning electron microscope (SEM) image in Figure 2c.

### 2.2 PDRC Performance and Evaluation

The optical properties of the DIW‐printed PZO textile are presented in Figure 2d,e. The textile exhibits a high solar reflectance of 96% normalized over the 0.3 – 2 µm wavelength range, along with a strong mid‐infrared emissivity of 97%, normalized within the 8 – 13 µm atmospheric window. For comparison, a pure PDMS film has been evaluated as a control. Although the PDMS film shows a comparable mid‐IR emissivity due to the intrinsic vibrational absorption of the polymer matrix, its solar reflectance is substantially lower because of the absence of ZrO_2_ nanoparticles. As a result, the exceptional optical characteristics of the PZO textile enable effective suppression of solar heat gain while simultaneously enhancing radiative heat dissipation from the human body to the surrounding environment. Remarkably, these combined optical properties surpass those of most previously reported integrated PDRC‐TENG textiles, as summarized in Figure S2. To further evaluate the practical reliability of the DIW‐printed textile under real‐world usage conditions, washability tests have been conducted by measuring the solar reflectance and infrared emissivity after 5 and 10 washing cycles. Despite detergent exposure and mechanical abrasion, the optical properties remain largely unchanged, demonstrating the robust stability of the printed textile (Figure S3). The outdoor radiative cooling performance of the DIW‐printed textile has been evaluated using the experimental setup depicted in Figure S4. The textile is placed on a flexible rubber heater simulating human skin with metabolic heat generation and tested under direct sunlight alongside a bare skin simulator, cotton fabric, and Ag fabric for comparison. As shown in Figure 2f, under active heating conditions relevant to personal thermal management, the DIW‐printed textile achieves temperature reductions of 6°C–10°C, 6°C–14°C, and 10°C–15°C relative to cotton fabric, bare skin, and Ag fabric, respectively. When the heater is turned off to eliminate simulated metabolic heat input, representing commonly used passive radiative cooling test conditions, the textile further exhibits sub‐ambient cooling of 3°C–6°C (Figure S5).

To further support the observed outdoor radiative cooling behavior, theoretical cooling power analysis was conducted using the hourly solar irradiance and ambient temperature records from Champaign, Illinois, on the day of the outdoor measurement (Figure S6). The sub‐ambient cooling temperature was calculated over a range of non‐radiative heat transfer coefficients (h_c_ = 2 – 10 W m^−^^2^ K^−^^1^) to account for different convective conditions in the outdoor environment. As shown in Figure S6a, the calculated temperature of the PZO textile reaches approximately 7°C below ambient at h_c_ = 6 W m^−2^ K^−1^. In addition, the net cooling power was calculated to be 79 W m^−^^2^ at 12 pm, when the solar intensity reached its peak (Figure S6b), confirming the substantial passive cooling capacity of the PZO textile under realistic outdoor conditions. These results are also consistent with the infrared thermal imaging in Figure 2g, which shows that the DIW‐printed textile remains significantly cooler than cotton fabric under sunlight exposure, highlighting its effectiveness in mitigating outdoor heat stress.

### 2.3 TENG Energy Harvesting and Motion Sensing

**FIGURE 3:**
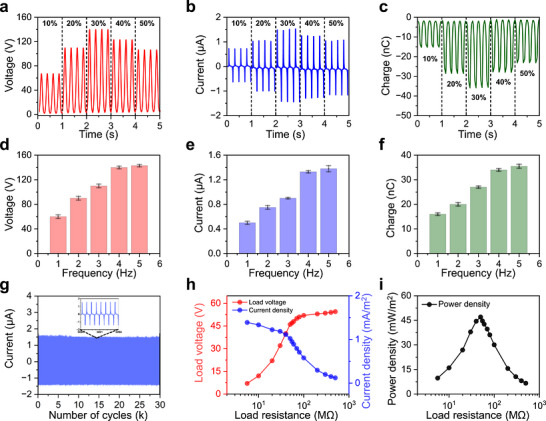
Triboelectric output performance of the PZO textile under various operating conditions. Dependence of (a) output voltage, (b) current, and (c) charge on ZrO_2_ concentrations (10–50 wt.%), measured at 4 Hz and 8 N. Effect of excitation frequency (1–5 Hz) on (d) output voltage, (e) current, and (f) charge at 30 wt.% ZrO_2_ under an applied force of 8 N. (g) Durability evaluation over 30 000 mechanical cycles, demonstrating stable output current. The inset shows representative current peaks observed across a few consecutive cycles. (h) Variation of load voltage and current density with external load resistance. (i) Corresponding power density as a function of load resistance.

The triboelectric performance of the DIW‐printed PZO textile has been evaluated by integrating it with an Ag fabric electrode to form a textile‐based TENG device. Operating in a contact–separation mode, the PZO nanocomposite acts as a triboelectrically negative layer owing to its high electron affinity, generating alternating electrical output under repeated contact with human skin (Figure S7). Systematic optimization reveals a pronounced dependence of the triboelectric output on ZrO_2_ loading, confirming its dual role in simultaneously enhancing optical scattering for radiative cooling and dielectric polarization for triboelectric charge generation. Increasing the ZrO_2_ concentration from 10 to 30 wt.% (Figure 3a–c) progressively enhances the electrical output, reaching peak values of 140 V, 1.33 µA, and 34 nC at 30 wt.%. This enhancement is attributed to the increased electron‐withdrawing capability and dielectric polarization introduced by ZrO_2_ within the PDMS matrix. Further increasing the ZrO_2_ content beyond 30 wt.% leads to a slight performance decline due to increased nanoparticle aggregation.

Under the optimized composition (30 wt.% ZrO_2_), the device approaches maximum electrical output under mechanical excitation at ∼4 Hz and 8 N, with output plateauing at higher frequencies and forces (Figure 3d–f and Figure S8). This observation is consistent with contact‐separation dynamics and charge saturation effects. Notably, the textile maintains stable performance over 30000 cycles, demonstrating excellent mechanical durability (Figure 3g). To evaluate practical power delivery capability, load matching analysis (Figure 3h) identifies an optimal resistance of 50 MΩ, yielding a peak power density of 47 mW m^−^^2^ (Figure 3i).

**FIGURE 4:**
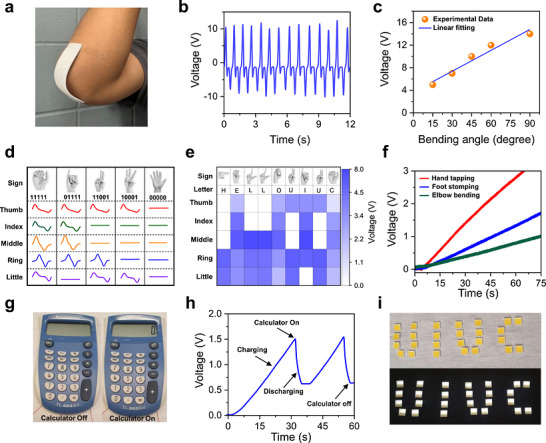
Demonstration of the PZO textile for motion sensing and energy harvesting. (a) Photograph of PZO textile attached to the elbow, showing conformal contact for motion detection. (b) Real‐time voltage signals generated during repeated elbow bending and extension. (c) Linear relationship between bending angle and output voltage. (d) Voltage responses from finger‐mounted PZO‐TENG sensors, enabling gesture recognition through binary signal encoding. (e) Voltage map of individual finger sensors corresponding to letters in American Sign Language. (f) Voltage outputs generated under various biomechanical motions, including hand tapping, foot stomping, and elbow bending. (g) Photographs of a calculator before and after being powered by the PZO‐TENG via mechanical stimulation. (h) Capacitor voltage profile illustrating charging by the PZO‐TENG and subsequent discharging while powering the calculator. (i) Illumination of 26 LEDs arranged to display “UIUC” on fabric, powered by the PZO‐TENG through repeated tapping.

The electrical output generated by the PZO‐TENG textile under mechanical stimulation enables both energy harvesting and self‐powered motion sensing. When attached to the elbow, the textile maintains stable operation under repeated bending, with output voltage tracking joint motion and increasing linearly with bending angle from 15° to 90°, demonstrating high sensitivity to contact area variation (Figure 4a–c). Extending this sensing capability, five finger‐mounted PZO‐TENG textile sensors enable real‐time monitoring of hand configurations (Figure S9). Binary encoding of finger bending (“1” for bent, “0” for unbent) allows reliable identification of distinct gestures (Figure 4d), while more nuanced finger positions corresponding to American Sign Language letters have been also resolved, as illustrated by voltage maps spelling “HELLO UIUC” (Figure 4e). These results demonstrate the applicability of PZO textile as a mechanically compliant, self‐powered sensing platform for wearable human‐machine interfaces, prosthetics, robotics, and assistive communication technologies.

For energy harvesting, the PZO‐TENG textile is evaluated under daily biomechanical motions, including tapping, clapping, and stomping at the wrist, palm, and foot, respectively (Figure 4f). As a representative demonstration, a 4.7 µF capacitor has been charged to ∼3 V within 60 s of hand tapping, sufficient to power small electronics such as a calculator and stopwatch (Figure 4g,h, and Figure S10) and to illuminate 26 series‐connected LEDs (Figure 4i and Movie S1). Benchmarking against recently reported PDRC‐TENG integrated textiles (Table S1) confirms that this triboelectric performance is achieved without compromising radiative cooling efficiency. Notably, the DIW‐printed PZO textile exhibits the highest reported solar reflectance and mid‐infrared emittance among integrated PDRC‐TENG systems, demonstrating its unique ability to synergistically combine efficient energy harvesting and high‐performance radiative cooling within a single breathable textile platform.

### 2.4 TENG‐based Motion‐Controlled Thermoregulation System

**FIGURE 5:**
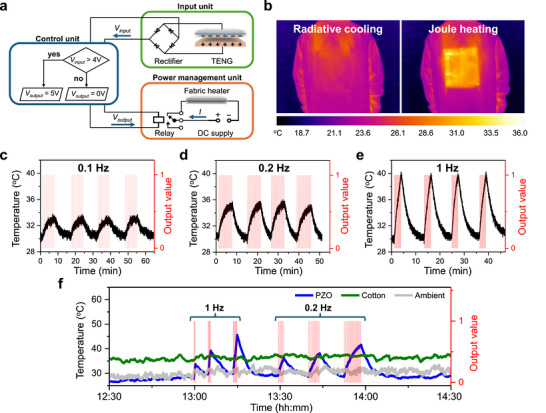
Motion‐governed thermoregulation integrating passive radiative cooling and on‐demand active heating. (a) Schematic of the motion‐triggered thermoregulation control system, in which the triboelectric output is rectified and processed by a control unit that activates a relay switch to power the fabric heater when the input voltage exceeds 4 V. (b) Infrared thermal images of the PZO textile operating in passive radiative cooling mode (left, no Joule heating) and active Joule heating mode (right) under shaded conditions. (c–e) Indoor temperature regulation under hand tapping at frequencies of 0.1, 0.2, and 1.0 Hz, respectively. The red‐shaded regions indicate heater activation triggered by TENG signals. (f) Outdoor thermoregulation demonstration comparing the PZO textile with cotton fabric at varying tapping frequencies and durations, illustrating motion‐controlled active heating integrated with passive radiative cooling.

To realize intelligent, activity‐responsive personal thermal control, we integrate the PDRC and TENG functionalities of the PZO textile with the underlying Ag fabric as a resistive heating layer to construct a closed‐loop wearable thermoregulation system (Figure 5a). In this design, biomechanical motion directly serves as the control input, generating triboelectric signals from the PZO textile that are rectified to activate on‐demand Joule heating in the Ag fabric, whereas in the absence of motion the textile autonomously maintains a passive radiative cooling state. Biomechanical activity was selected as the control trigger because the PZO textile intrinsically integrates TENG functionality within its single‐layer nanocomposite matrix. Furthermore, this self‐generated triboelectric signal naturally reflects the user's activity state and real‐time thermoregulatory demand under wearable conditions. In practical scenarios like outdoor commuting or walking, supplemental heating is typically required intermittently rather than continuously. This mechanism allows the textile to maintain passive cooling during rest while activating Joule heating only during active motion. Consequently, it enables adaptive, activity‐responsive thermal regulation without external sensors or manual switches. When the motion‐induced voltage exceeds a preset threshold, the control circuit switches the system from passive cooling to active heating, enabling seamless and reversible thermal mode transitions. Infrared thermal imaging confirms this dynamic behavior, showing rapid temperature elevation upon mechanical activation compared to the passive state (Figure 5b). Note that these thermal images were recorded under shaded conditions to simulate a cold environment. Due to the absence of direct solar loading, the baseline thermal contrast between the PZO textile and the surrounding shirt is less pronounced than in the outdoor sunlight tests (Figure 2g). Indoor evaluations at a fixed heating power of 0.23 W cm^−^^2^ demonstrate that the heating response is dynamically governed by hand tapping frequency and duration. Increasing the frequency from 0.1 to 0.2 Hz raises the textile temperature by 3°C and 5°C, respectively, over a 7 min duration (Figure 5c,d), while tapping at 1 Hz produced a 10°C increase within 3.8 min (Figure 5e). These results confirm that thermal output is effectively modulated by mechanical input frequency and duration, enabling user‐regulated heating through natural motion. To further optimize the thermoregulation system, heating power and output voltage duration were investigated (Figures S11 and S12). Here, the output voltage duration refers to the duration of voltage generation applied to the heater after each input voltage peak is detected. At a fixed hand‐tapping frequency of 2 Hz, the lower heating power of 0.06 W cm^−^^2^ increased the textile temperature only to approximately 28°C after 2 min, whereas the selected heating power of 0.23 W cm^−^^2^ increased it to approximately 33°C under the same condition (Figure S11). This confirms that the selected heating power is sufficient to approach the thermal comfort range within a practical activation period. The output voltage duration was also optimized to maintain motion‐dependent controllability. When the duration was fixed at 5 s for each detected input voltage peak, multiple subsequent peaks exceeding the threshold occurred during a single prolonged heating output, limiting fine control by tapping frequency (Figure S12). Therefore, a shorter output voltage duration of 1 s was selected to enable more responsive heat regulation according to biomechanical input.

Finally, outdoor thermoregulation tests further validate dual‐mode functionality under realistic conditions (Figure 5f). Compared with cotton fabric, which consistently exhibits higher temperatures under sunlight, the PZO textile maintains superior radiative cooling performance in passive cooling mode. Upon mechanical activation, the heating response scales with tapping frequency and duration. As illustrated in the figure, a higher tapping frequency (1 Hz) triggers a rapid and sharp temperature rise, whereas a lower frequency (0.2 Hz) results in a more gradual thermal response. In both regimes, the magnitude of the temperature increase (ΔT) is proportional to the tapping duration. These results confirm motion‐programmable heating while preserving efficient daytime radiative cooling, enabling adaptive thermal management in dynamic outdoor environments. This architecture establishes a motion‐governed thermoregulatory textile that intrinsically couples sensing, energy harvesting, and adaptive thermal control within a single material platform. Future developments are expected to further enhance the wearable applicability of the system through the incorporation of sweat transport pathways, improved durability under environmental aging, and additional performance enhancement strategies for long‐term adaptive wearable applications.

## 3 Conclusion

In summary, we develop a fiber‐structured multifunctional textile via DIW that intrinsically integrates passive daytime radiative cooling, triboelectric energy harvesting, self‐powered sensing, and motion‐triggered heating within a single nanocomposite platform. Central to this design is the dual‐function role of ZrO_2_, which simultaneously enhances solar scattering for high reflectance (96%) and dielectric polarization for amplified triboelectric output, enabling concurrent optimization of optical and electrical performance within one material system. This single‐step, programmable fabrication of fiber architecture preserves breathability and mechanical compliance, while overcoming the structural complexity and trade‐offs of conventional multilayer designs. Furthermore, motion‐induced triboelectric signals directly actuate Joule heating, enabling seamless switch between passive cooling and active heating under dynamic conditions. This motion‐controlled thermoregulatory capability demonstrates a scalable and unified material platform for next‐generation smart textiles that combine adaptive thermal management, energy autonomy, and wearable intelligence.

## 4 Experimental Section

### 4.1 Sample Fabrication

The PDMS elastomer base and curing agent (SE1700 base, Dow Corning Corporation) were mixed at a 10:1 mass ratio. To prepare the PZO ink, ZrO_2_ nanoparticles (US Research Nanomaterials, Inc.) were added to the PDMS mixture at a PDMS:ZrO_2_ weight ratio of 10:3, followed by speed mixing (FlackTek 330‐100 SE) at 2000 rpm for 2 min.

### 4.2 Direct Ink Writing (DIW) Process

DIW was performed at room temperature using a customized printer (AGS‐1000, Aerotech Inc.). Pattern geometries were designed in AutoCAD and converted to G‐code using CADFusion. A pneumatic dispenser extruded the ink through a tapered nozzle onto the substrate. After printing, samples were cured either at room temperature for 24 h or in an oven at 65°C for 6 h to crosslink and solidify the printed textile. For TENG fabrication, PZO ink was printed directly onto silver‐coated fabric serving as the conductive electrode.

### 4.3 Optical Characterization and Morphology

Spectral reflectance of the DIW‐printed PZO textile in the visible and near‐infrared range was assessed using a UV–vis–near infrared spectrometry (Cary 5000, Agilent). Emissivity in the mid‐infrared wavelength range was measured using FTIR spectrometry (Thermo Nicolet iS50). SEM images were acquired with HitachiS‐4800 scanning electron microscope, and thermal infrared images and videos were captured using a Fluke TiS75+ Thermal Imager.

### 4.4 Indoor TENG‐Actuated Thermoregulation Test

A feedback control system was constructed to regulate the temperature of the PZO textile using its triboelectric output. The system consisted of the PZO textile, an Arduino microcontroller, a solid‐state relay, and a DC power supply (Keithley 2260B‐250‐4). A K‐type thermocouple (Omega Engineering) was positioned beneath the PZO textile on a Styrofoam support to monitor temperature in real‐time. Both the rectified TENG output and the thermocouple signal were recorded through the Arduino. To activate Joule heating at a power density of 10 W m^−2^, hand tapping was applied to the PZO textile at varying frequencies. Given the high voltage and short duration of the rectified TENG output, the Arduino was programmed to output a 5 V control signal to the relay for 1 s whenever the input voltage exceeded 4 V. Each hand‐tapping cycle lasted 7 min, with four cycles repeated to demonstrate the repeatability.

### 4.5 Outdoor Radiative Cooling and TENG‐Actuated Thermoregulation Tests

For outdoor testing, a flexible rubber heater (Omega Engineering) was used as a skin simulator to replicate human metabolic heat generation, powered by a DC power supply (Keithley 2260B‐250‐4) to produce 80 W m^−2^, comparable to typical human metabolic heat flux. A K‐type thermocouple was attached to the skin simulator to monitor its temperature, which was recorded via a USB data acquisition device (USB‐TC, Measurement Computing). For the outdoor radiative cooling tests, multiple skin simulators covered with different textile samples were mounted on a Styrofoam support wrapped with Mylar film. The cooling performance of the PZO textile was compared with that of Ag‐coated fabric, a bare heater (simulating uncovered skin), and commercial white cotton fabric. For motion‐controlled thermoregulation demonstration during the outdoor test, the same feedback control system used in indoor testing was applied. Outdoor experiments were conducted on August 2nd, 2024, in Urbana, Illinois, under direct sunlight.

### 4.6 Electrical Measurement

The TENG output current of the PZO textile was measured using a Keithley 6514 electrometer (Keithley, Cleveland, OH, USA). Voltage output was characterized using a P6015A high‐voltage probe connected to a TBS1052C oscilloscope (Tektronix, Beaverton, OR, USA). Electrical signals corresponding to human movement and finger motion were recorded using the oscilloscope.

### 4.7 Water Vapor Permeation Test

A plastic jar was filled with water, and the lid was modified with a 4.8 cm diameter opening to allow for water vapor transmission. The textile sample was placed between the jar and the lid, with a rubber O‐ring inserted to ensure an airtight seal. The assembly was stored at 20°C and 16% relative humidity, and water loss was monitored over time.

### 4.8 Washability Test

A mini washing machine (DTOWER 6.5L Folding) was used with Alconox detergent powder at a water temperature of 30°C. The DIW‐printed textile was placed into the machine along with 4 grams of detergent per liter of water. One washing cycle consisted of 5 min of spinning. Spectral characterization was performed before and after 5 and 10 washing cycles.

## Author Contributions

L.C. conceived the idea. Y.Y.C. carried out the SEM characterization, thermal imaging, and adaptive thermoregulation experiments. P.K. performed DIW fabrication, optical characterization, and outdoor thermal measurements. M.S. conducted TENG characterizations, and energy harvesting demonstrations. L.C. supervised and directed the project. L.C., Y.Y.C., P.K. and M.S. wrote the manuscript. All authors reviewed and commented on the paper.

## Conflicts of Interest

L.C. is a co‐founder of SolarMantle Inc. and an inventor on patents related to radiative cooling technologies. The remaining authors declare no competing interests.

## Supporting information




**Supporting File 1**: advs76121‐sup‐0001‐SuppMat.docx.

**Supporting File 2**: advs76121‐sup‐0002‐VideoS1.mp4.

## Data Availability

The data that support the findings of this study are available from the corresponding author upon reasonable request.
