# Supplementary material for: Intrinsic Coupling of Radiative Cooling and Triboelectric Responses in Dual‐Function ZrO2 Nanocomposites for Adaptive Thermoregulation
Source: Adv Sci (Weinh). 2026 Jun 15;13(51):e76121. doi: 10.1002/advs.76121 (PMC13336401; doi:10.1002/advs.76121)
Supplement: Supplementary file 1 [file ADVS-13-e76121-s002.docx]

**Intrinsic Coupling of Radiative Cooling and Triboelectric Responses in Dual-Function ZrO_2_ Nanocomposites for Adaptive Thermoregulation**

Yoon Young Choi,^1^ Pranto Karua,^1^ Md Salauddin,^1^ Lili Cai^1,2,^*

^1^Department of Mechanical Science and Engineering, the Grainger College of Engineering, University of Illinois Urbana-Champaign, Urbana, IL 61801, United States

^2^Materials Research Laboratory, the Grainger College of Engineering, University of Illinois at Urbana−Champaign, Urbana, IL 61801, United States

*Corresponding author email: [lilicai@illinois.edu](mailto:lilicai@illinois.edu)

The PDF file includes:

- Supplementary Texts
- Supplementary Figures S1 to S12
- Supplementary Table S1
- References

**Supplementary Text**

## **Solar reflectance calculation:**

The weighted solar reflectance was calculated using the following formula:

$$R=\frac{\int_{0.3 \mu m}^{2 \mu m} R_{solar}(\lambda)*I_{AM1.5}(\lambda) d\lambda}{\int_{0.3 \mu m}^{2 \mu m} I_{AM1.5}(\lambda) d\lambda}$$

where $R$ is the weighted average solar reflectance, $\lambda$ is the wavelength, $R_{solar}(\lambda)$ is the spectral solar reflectance, and $I_{AM1.5}(\lambda)$ is the solar spectral intensity based on the Air Mass 1.5 Global (AM1.5G) standard.

## **Infrared emissivity calculation:**

The weighted infrared emissivity in the 8 – 13 μm range was calculated using the following formula:

$$\epsilon=\frac{\int_{8 \mu m}^{13 \mu m} \epsilon(\lambda)*I_{BB,300K}(\lambda) d\lambda}{\int_{8 \mu m}^{13 \mu m} I_{BB,300K}(\lambda) d\lambda}$$

where $\epsilon$ is the weighted average infrared emissivity, $\lambda$ is the wavelength, $I_{BB,300K}(\lambda)$ is the spectral blackbody intensity at 300 K (~27°C).

## **Cooling power calculation:**

The net cooling power ($P_{\mathrm{cool}}$) of the PZO textile is determined by the balance between the power radiated by the textile and the heat gains from the outdoor environment, including absorbed solar irradiance ($P_{\mathrm{sun}}$), atmospheric thermal radiation ($P_{\mathrm{atm}}$), and non-radiative parasitic heat gain ($P_{\mathrm{nonrad}}$) via conduction and convection. The governing equation is expressed as:

$$P_{\mathrm{cool}}\left( T \right)=P_{\mathrm{rad}}\left( T \right)-P_{\mathrm{sun}}-P_{\mathrm{atm}}\left( T_{\mathrm{amb}} \right)-P_{\mathrm{nonrad}}$$

where T is the surface temperature of the PZO textile and T_amb_ is the ambient air temperature. Each term is defined as follows:

$$P_{\mathrm{rad}}\left( T \right)=\int d\Omega cos\theta\int_{0}^{\infty} d\lambda I_{\mathrm{BB}}\left( T,\lambda\right)\varepsilon\left( \lambda\right)$$

$$P_{\mathrm{sun}}=\int_{0}^{\infty} d\lambda I_{AM1.5}\left( \lambda\right)\alpha\left( \lambda\right)$$

$$P_{\mathrm{atm}}\left( T_{\mathrm{amb}} \right)= \int d\Omega cos\theta\int_{0}^{\infty} d\lambda I_{\mathrm{BB}}\left( T_{\mathrm{amb}},\lambda\right)\left( 1-{t_{\mathrm{atm}}\left( \lambda\right)}^{1/cos\theta} \right) \alpha\left( \lambda\right)$$

$$P_{\mathrm{nonrad}}\left( {T,T}_{\mathrm{amb}} \right)=h_{c}\left( T_{\mathrm{amb}}-T \right)$$

In these equations, α is the absorptivity, and t_atm_ is the atmospheric transmittance at the zenith. θ is the zenith angle, I_AM1.5_ is the standard solar intensity spectrum, and I_BB_ is the spectral radiance of a blackbody. h_c_ denotes the combined non-radiative heat transfer coefficient for conduction and convection.

For this analysis, we assumed the PZO textile faces the zenith ($\theta=0$) and exhibits diffuse absorptivity/emissivity. The values for P_rad_ and P_sun_ were derived using the measured UV-Vis-NIR and FTIR spectra of the PZO textile. To calculate P_atm_, we utilized hourly solar irradiance and ambient temperature data for Champaign, IL, obtained from the NASA POWER database.^[49]^ The net cooling power, P_cool_, was calculated at $T=T_{\mathrm{amb}}$. Under this condition, P_cool_ quantifies the intrinsic cooling capacity of the PZO textile under the given outdoor environment. A positive value indicates that radiative heat dissipation exceeds the combined heat gains from solar absorption, atmospheric radiation, and non-radiative heat transfer, resulting in net cooling of the textile.

**Supplementary Figures**

**Figure S1.** Water vapor permeation of the DIW-printed PZO and cotton textile for three consecutive days.

**Figure S2.** Comparison of solar reflectance and mid-infrared emissivity/transmittance of the DIW-printed PZO textile with recently reported radiative cooling textiles integrated with TENG functions.

**Figure S3.** (a) Solar reflectance and (b) infrared emissivity spectra of the DIW-printed PZO textile before and after 5 and 10 wash cycles.

**Figure S4.** (a) Photograph and (b) schematic of the experimental setup for the outdoor thermal measurements.

**Figure S5.** (a) Measured sub-ambient cooling temperatures of the PZO textile under outdoor conditions. (b) Solar irradiance was recorded on August 2, 2024.

**Figure S6.** (a) Calculated subcooling temperatures of the PZO textile on a sunny day under different non-radiative heat transfer coefficients, h_c_. (b) Contributions of radiative cooling, solar absorption, atmospheric radiation, and non-radiative heat transfer to the net cooling power as a function of h_c_.

**Figure S7.** Schematic illustration of the triboelectric charge generation mechanism involving the DIW-printed PZO textile, Ag-coated fabric electrode, and human skin.

**Figure S8.** (a) Output voltage, (b) current, and (c) charge generated from the PZO-TENG device under different excitation forces.

**Figure S9.** Schematic illustration of finger-mounted PZO-TENG textile sensors to monitor finger bending for real-time gesture recognition.

**Figure S10.** (a) Demonstration of a stopwatch powered by the PZO-TENG, and (b) corresponding capacitor charging–discharging cycles during the stopwatch ON/OFF operation.

**Figure S11.** Comparison of temperature increases under different heating powers. The hand tapping frequency was fixed at 2Hz.

 **Figure S12.** Input voltage generated by hand tapping and the corresponding output value of the thermoregulation control system when the heating duration (output voltage duration) was fixed at 5 s for each detected input voltage signal. The blue dotted line indicates the threshold for triggering the output voltage.

**Supplementary Table**

**Table S1.** Comparison of solar reflectance and mid-infrared emissivity of recently reported daytime radiative cooling materials integrated with triboelectric nanogenerator functionalities

| **Composition** | ***Solar reflectance (%)*** | ***IR emissivity/transmittance (%)*** | **References** |
| --- | --- | --- | --- |
| Bilayer SiO_2_/PVA and the PVDF-TrFE@SEBS | 0.95 | 0.81 | [37] |
| PAN/BT and PAN/CB | 0.90 | 0.95 | [38] |
| PVDF-HFP/SiO_2_ and LM-Ag-SEBS | 0.83 | 0.95 | [41] |
| PAA-Zn/ZnO and PTFE | 0.89 | 0.94 | [42] |
| Ecoflex@h-BN / Al | 0.92 | 0.97 | [43] |
| P(VDF-HFP) and SiO_2_/ZnO/ZIF-8 | 0.88 | 0.95 | [44] |
| TiO_2_ nfs@CA t-TENGs | 0.93 | 0.85 | [45] |
| PE/UV/OM-CF | 0.90 | 0.76 | [46] |
| Ecoflex@BTO@UAFL | 0.94 | 0.97 | [50] |
| Ta_2_O_5_/TPU | 0.90 | 0.83 | [51] |
| PVDF-HFP nano fiber | 0.95 | 0.91 | [52] |
| DIW-printed PZO textile | 0.96 | 0.97 | This Work |

**References**

1. D. Shou, Z. Li, Sustainable personal cooling in a warming world, *Science* **2025**, 389, 877, <https://doi.org/10.1126/science.adt9536>.

2. L. Lei, S. Shi, D. Wang, S. Meng, J.-G. Dai, S. Fu, J. Hu, Recent advances in thermoregulatory clothing: materials, mechanisms, and perspectives, *ACS nano* **2023**, 17, 1803, <https://doi.org/10.1021/acsnano.2c10279>.

3. Y. Jung, M. Kim, T. Kim, J. Ahn, J. Lee, S. H. Ko, Functional materials and innovative strategies for wearable thermal management applications, *Nano-Micro Letters* **2023**, 15, 160, <https://doi.org/10.1007/s40820-023-01126-1>.

4. I. Staffell, S. Pfenninger, N. Johnson, A global model of hourly space heating and cooling demand at multiple spatial scales, *Nature Energy* **2023**, 8, 1328, <https://doi.org/10.1038/s41560-023-01341-5>.

5. Y. Peng, Y. Cui, Advanced textiles for personal thermal management and energy, *Joule* **2020**, 4, 724, <https://doi.org/10.1016/j.joule.2020.02.011>.

6. J. Chai, Z. Kang, Y. Yan, L. Lou, Y. Zhou, J. Fan, Thermoregulatory clothing with temperature-adaptive multimodal body heat regulation, *Cell Reports Physical Science* **2022**, 3, <https://doi.org/10.1016/j.xcrp.2022.100958>.

7. X. Lan, Y. Wang, J. Peng, Y. Si, J. Ren, B. Ding, B. Li, Designing heat transfer pathways for advanced thermoregulatory textiles, *Materials Today Physics* **2021**, 17, 100342, <https://doi.org/10.1016/j.mtphys.2021.100342>.

8. A. P. Raman, M. A. Anoma, L. Zhu, E. Rephaeli, S. Fan, Passive radiative cooling below ambient air temperature under direct sunlight, *Nature* **2014**, 515, 540, <https://doi.org/10.1038/nature13883>.

9. J. Mandal, Y. Fu, A. C. Overvig, M. Jia, K. Sun, N. N. Shi, H. Zhou, X. Xiao, N. Yu, Y. Yang, Hierarchically porous polymer coatings for highly efficient passive daytime radiative cooling, *Science* **2018**, 362, 315, <https://doi.org/10.1126/science.aat9513>.

10. A. Leroy, B. Bhatia, C. C. Kelsall, A. Castillejo-Cuberos, M. Di Capua H, L. Zhao, L. Zhang, A. Guzman, E. Wang, High-performance subambient radiative cooling enabled by optically selective and thermally insulating polyethylene aerogel, *Science advances* **2019**, 5, eaat9480, <https://doi.org/10.1126/sciadv.aat9480>.

11. X. Xue, M. Qiu, Y. Li, Q. Zhang, S. Li, Z. Yang, C. Feng, W. Zhang, J. G. Dai, D. Lei, Creating an eco‐friendly building coating with smart subambient radiative cooling, *Adv Mater* **2020**, 32, 1906751, <https://doi.org/10.1002/adma.201906751>.

12. K. Zhou, W. Li, B. B. Patel, R. Tao, Y. Chang, S. Fan, Y. Diao, L. Cai, Three-dimensional printable nanoporous polymer matrix composites for daytime radiative cooling, *Nano letters* **2021**, 21, 1493, <https://doi.org/10.1021/acs.nanolett.0c04810>.

13. D. Li, X. Liu, W. Li, Z. Lin, B. Zhu, Z. Li, J. Li, B. Li, S. Fan, J. Xie, Scalable and hierarchically designed polymer film as a selective thermal emitter for high-performance all-day radiative cooling, *Nature Nanotechnology* **2021**, 16, 153, <https://doi.org/10.1038/s41565-020-00800-4>.

14. J. Park, H. Lim, H. Keawmuang, D. Chae, H. Lee, J. Rho, Flexible Self‐Cleaning Janus Emitter for Transparent Radiative Cooling in Enclosed Spaces, *Small* **2025**, 21, 2501840.

15. H. Keawmuang, T. Badloe, C. Lee, J. Park, J. Rho, Inverse design of colored daytime radiative coolers using deep neural networks, *Solar Energy Materials and Solar Cells* **2024**, 271, 112848.

16. M. Lee, G. Kim, Y. Jung, K. R. Pyun, J. Lee, B.-W. Kim, S. H. Ko, Photonic structures in radiative cooling, *Light: Science & Applications* **2023**, 12, 134.

17. H. Keawmuang, J. Park, H. Lee, S. Choi, S. So, C. Lim, T. Badloe, C. Hyeon, M. Jeong, D. Lee, Recent Advances in Radiative Cooling: From Fundamentals to Commercial Applications, *ACS Applied Materials & Interfaces* **2026**.

18. P.-C. Hsu, A. Y. Song, P. B. Catrysse, C. Liu, Y. Peng, J. Xie, S. Fan, Y. Cui, Radiative human body cooling by nanoporous polyethylene textile, *Science* **2016**, 353, 1019, <https://doi.org/10.1126/science.aaf5471>.

19. X. Wu, J. Li, Q. Jiang, W. Zhang, B. Wang, R. Li, S. Zhao, F. Wang, Y. Huang, P. Lyu, An all-weather radiative human body cooling textile, *Nature Sustainability* **2023**, 6, 1446, <https://doi.org/10.1038/s41893-023-01200-x>.

20. P.-C. Hsu, C. Liu, A. Y. Song, Z. Zhang, Y. Peng, J. Xie, K. Liu, C.-L. Wu, P. B. Catrysse, L. Cai, A dual-mode textile for human body radiative heating and cooling, *Science advances* **2017**, 3, e1700895, <https://doi.org/10.1126/sciadv.1700895>.

21. L. Cai, A. Y. Song, W. Li, P. C. Hsu, D. Lin, P. B. Catrysse, Y. Liu, Y. Peng, J. Chen, H. Wang, Spectrally selective nanocomposite textile for outdoor personal cooling, *Adv Mater* **2018**, 30, 1802152, <https://doi.org/10.1002/adma.201802152>.

22. L. Cai, A. Y. Song, P. Wu, P.-C. Hsu, Y. Peng, J. Chen, C. Liu, P. B. Catrysse, Y. Liu, A. Yang, Warming up human body by nanoporous metallized polyethylene textile, *Nature communications* **2017**, 8, 496, <https://doi.org/10.1038/s41467-017-00614-4>.

23. Y. Y. Choi, K. Zhou, H. K. Woo, D. Patel, M. Salauddin, L. Cai, Radiative cooling smart textiles with integrated sensing for adaptive thermoregulation, *ACS Materials Letters* **2024**, 6, 4624, <https://doi.org/10.1021/acsmaterialslett.4c01624>.

24. K. Zhou, S. Tang, P. Karua, F. Wu, S. Hong, D. Patel, G. M. Reeser, D. M. Cropek, P. V. Braun, L. Cai, Printable polymer nanocomposites for scalable and architected radiative cooling, *Nature Communications* **2025**, <https://doi.org/10.1038/s41467-025-67831-0>.

25. S. Suhendri, M. Hu, Y. Dan, Y. Su, B. Zhao, S. Riffat, Building energy-saving potential of a dual-functional solar heating and radiative cooling system, *Energy and Buildings* **2024**, 303, 113764, <https://doi.org/10.1016/j.enbuild.2023.113764>.

26. X. Li, B. Sun, C. Sui, A. Nandi, H. Fang, Y. Peng, G. Tan, P.-C. Hsu, Integration of daytime radiative cooling and solar heating for year-round energy saving in buildings, *Nature communications* **2020**, 11, 6101, <https://doi.org/10.1038/s41467-020-19790-x>.

27. S. Xue, G. Huang, Q. Chen, X. Wang, J. Fan, D. Shou, Personal thermal management by radiative cooling and heating, *Nano-micro letters* **2024**, 16, 153.

28. R. Zhang, Y. Li, B. Yao, M. Chen, An electrospinning flexible textile integrating radiative cooling and solar heating for dynamic thermoregulation, *Journal of Materials Chemistry A* **2025**, 13, 38878.

29. T.-H. Chen, P.-C. Hsu, Wearable variable-emittance devices—The future of dynamic personal thermoregulation, *Applied Physics Letters* **2024**, 125.

30. N. Guo, L. Yu, C. Shi, H. Yan, M. Chen, A facile and effective design for dynamic thermal management based on synchronous solar and thermal radiation regulation, *Nano Letters* **2024**, 24, 1447.

31. F.-R. Fan, Z.-Q. Tian, Z. L. Wang, Flexible triboelectric generator, *Nano energy* **2012**, 1, 328, <https://doi.org/10.1016/j.nanoen.2012.01.004>.

32. Z. L. Wang, Triboelectric nanogenerators as new energy technology for self-powered systems and as active mechanical and chemical sensors, *ACS nano* **2013**, 7, 9533, <https://doi.org/10.1021/nn404614z>.

33. S. Wang, Y. Xie, S. Niu, L. Lin, Z. L. Wang, Freestanding triboelectric‐layer‐based nanogenerators for harvesting energy from a moving object or human motion in contact and non‐contact modes, *Adv Mater* **2014**, 26, 2818, <https://doi.org/10.1002/adma.201305303>.

34. Y. Wang, Y. Yang, Z. L. Wang, Triboelectric nanogenerators as flexible power sources, *npj Flexible Electronics* **2017**, 1, 10, <https://doi.org/10.1038/s41528-017-0007-8>.

35. S. S. Kwak, H. J. Yoon, S. W. Kim, Textile‐based triboelectric nanogenerators for self‐powered wearable electronics, *Advanced Functional Materials* **2019**, 29, 1804533, <https://doi.org/10.1002/adfm.201804533>.

36. Y. Yang, H. Zhang, Z.-H. Lin, Y. S. Zhou, Q. Jing, Y. Su, J. Yang, J. Chen, C. Hu, Z. L. Wang, Human skin based triboelectric nanogenerators for harvesting biomechanical energy and as self-powered active tactile sensor system, *ACS nano* **2013**, 7, 9213, <https://doi.org/10.1021/nn403838y>.

37. Y. Peng, H. Huang, H. Liu, J. Dong, Y. Zhang, J. Long, Y. Huang, Robust Triboelectric E-Textile with Semi-bonded Bilayers for On-Skin Thermal Regulation and Self-Powered Motion Monitoring, *Adv Fiber Mater* **2025**, 1, <https://doi.org/10.1007/s42765-025-00546-5>.

38. G. Ye, Y. Wan, J. Wu, W. Zhuang, Z. Zhou, T. Jin, J. Zi, D. Zhang, X. Geng, P. Yang, Multifunctional device integrating dual-temperature regulator for outdoor personal thermal comfort and triboelectric nanogenerator for self-powered human-machine interaction, *Nano Energy* **2022**, 97, 107148, <https://doi.org/10.1016/j.nanoen.2022.107148>.

39. W. Ou-Yang, L. Liu, M. Xie, S. Zhou, X. Hu, H. Wu, Z. Tian, X. Chen, Y. Zhu, J. Li, Recent advances in triboelectric nanogenerator-based self-powered sensors for monitoring human body signals, *Nano Energy* **2024**, 120, 109151, <https://doi.org/10.1016/j.nanoen.2023.109151>.

40. J. Yi, K. Dong, S. Shen, Y. Jiang, X. Peng, C. Ye, Z. L. Wang, Fully fabric-based triboelectric nanogenerators as self-powered human–machine interactive keyboards, *Nano-micro letters* **2021**, 13, 103, <https://doi.org/10.1007/s40820-021-00621-7>.

41. C. Fan, Z. Long, Y. Zhang, A. Mensah, H. He, Q. Wei, P. Lv, Robust integration of energy harvesting with daytime radiative cooling enables wearing thermal comfort self-powered electronic devices, *Nano Energy* **2023**, 116, 108842, <https://doi.org/10.1016/j.nanoen.2023.108842>.

42. Z. Sun, Y. Hu, W. Wei, Y. Li, Q. Zhang, K. Li, H. Wang, C. Hou, Hyperstable eutectic core‐spun fiber enabled wearable energy harvesting and personal thermal management fabric, *Adv Mater* **2024**, 36, 2310102, <https://doi.org/10.1002/adma.202310102>.

43. S. Wang, Y. Wu, M. Pu, M. Xu, R. Zhang, T. Yu, X. Li, X. Ma, Y. Su, H. Tai, A versatile strategy for concurrent passive daytime radiative cooling and sustainable energy harvesting, *Small* **2024**, 20, 2305706, <https://doi.org/10.1002/smll.202305706>.

44. R. Liu, K. Xia, T. Yu, F. Gao, Q. Zhang, L. Zhu, Z. Ye, S. Yang, Y. Ma, J. Lu, Multifunctional smart fabrics with integration of self-cleaning, energy harvesting, and thermal management properties, *ACS nano* **2024**, 18, 31085, <https://doi.org/10.1021/acsnano.4c08324>.

45. Z. Chen, M. Xu, C. Zhou, Z. Hu, Z. Du, X. Fu, Y. Song, X. Wen, J. Wang, G. Cai, Phase transformation enabled textile triboelectric nanogenerators for wearable energy harvesting and personal thermoregulation, *Nano Energy* **2024**, 132, 110361, <https://doi.org/10.1016/j.nanoen.2024.110361>.

46. M. Cheng, X. Liu, Z. Li, Y. Zhao, X. Miao, H. Yang, T. Jiang, A. Yu, J. Zhai, Multiple textile triboelectric nanogenerators based on UV-protective, radiative cooling, and antibacterial composite yarns, *Chemical Engineering Journal* **2023**, 468, 143800, <https://doi.org/10.1016/j.cej.2023.143800>.

47. Y. Xie, S. Wang, S. Niu, L. Lin, Q. Jing, Y. Su, Z. Wu, Z. L. Wang, Multi-layered disk triboelectric nanogenerator for harvesting hydropower, *Nano Energy* **2014**, 6, 129, <https://doi.org/10.1016/j.nanoen.2014.03.015>.

48. P. Bai, G. Zhu, Z.-H. Lin, Q. Jing, J. Chen, G. Zhang, J. Ma, Z. L. Wang, Integrated multilayered triboelectric nanogenerator for harvesting biomechanical energy from human motions, *ACS nano* **2013**, 7, 3713, <https://doi.org/10.1021/nn4007708>.

49. NASA Langley Research Center. *POWER Data Access Viewer: Prediction of Worldwide Energy Resources*. National Aeronautics and Space Administration. Accessed Month Day, Year. [https://power.larc.nasa.gov/data-access-viewer](https://power.larc.nasa.gov/data-access-viewer/).

50. Y. Wu, S. Wang, R. Zhang, T. Yu, M. Xu, X. Li, M. Pu, X. Ma, Y. Guo, Y. Su, A novel multifunctional photonic film for colored passive daytime radiative cooling and energy harvesting, *Small* **2024**, 20, 2308661, <https://doi.org/10.1002/smll.202308661>.

51. X. Li, W. Qin, Y. Wang, Y. Zhang, Z. Tian, W. Li, X. Li, S. Yin, Recyclable Bead Chain Structure of Ta2O5/TPU Fiber Films for Energy Harvesting and Passive Daytime Radiative Cooling, *ACS Sustainable Chemistry & Engineering* **2024**, 12, 17167, <https://pubs.acs.org/doi/10.1021/acssuschemeng.4c05757>.

52. Y. Jung, J. Ahn, J. S. Kim, J. Bang, M. Kim, S. Jeong, J. Lee, S. Han, I. K. Oh, S. H. Ko, All Weather‐Usable Wearable Dual Energy Harvester for Outdoor Sustainable Operation, *SusMat* **2025**, 5, e264, <https://doi.org/10.1002/sus2.264>.
